# Crop insect pest detection based on dilated multi-scale attention U-Net

**DOI:** 10.1186/s13007-024-01163-w

**Published:** 2024-02-26

**Authors:** Xuqi Wang, Shanwen Zhang, Ting Zhang

**Affiliations:** School of Electronic Information, Xijing University, Xi’an, 710123 China

**Keywords:** Insect pest, Detection and segmentation, U-Net, Dilated inception, Multi-scale attention

## Abstract

**Background:**

Crop pests seriously affect the yield and quality of crops. Accurately and rapidly detecting and segmenting insect pests in crop leaves is a premise for effectively controlling insect pests.

**Methods:**

Aiming at the detection problem of irregular multi-scale insect pests in the field, a dilated multi-scale attention U-Net (DMSAU-Net) model is constructed for crop insect pest detection. In its encoder, dilated Inception is designed to replace the convolution layer in U-Net to extract the multi-scale features of insect pest images. An attention module is added to its decoder to focus on the edge of the insect pest image.

**Results:**

The experiments on the crop insect pest image IP102 dataset are implemented, and achieved the detection accuracy of 92.16% and IoU of 91.2%, which is 3.3% and 1.5% higher than that of MSR-RCNN, respectively.

**Conclusion:**

The results indicate that the proposed method is effective as a new insect pest detection method. The dilated Inception can improve the accuracy of the model, and the attention module can reduce the noise generated by upsampling and accelerate model convergence. It can be concluded that the proposed method can be applied to practical crop insect pest monitoring system.

## Introduction

Crop pests are a major agricultural problem in the world, which seriously affect the yield and quality of crops. Crop insect pest detection is the premise and foundation of crop insect pest identification and control [1, 2]. There are many crop insect pest detection methods [3, 4]. They are broadly divided into two categories: traditional machine learning (ML) [5] and deep learning (DL) [6–8]. YOLO, U-Net and their variants have been widely applied to crop insect pest detection task, and achieved remarkable results [9–11]. Galphat et al. [12] comprehensively reviewed and analyzed the algorithms and technologies of insect pest detection in the agricultural field. Domingues et al. [13] presented a literature review on ML and DL used in the agricultural sector, focusing on the tasks of classification, detection, and prediction of diseases and pests, with an emphasis on tomato crops. Liu et al. [14] summarized the research on plant pest detection based on DL in recent years from three aspects: classification network, detection network and segmentation network, introduced the advantages and disadvantages of each method, and discussed the challenges that DL-based crop pest detection may face in practical applications. One of the most essential and beneficial properties of DL is its ability to generate features autonomously [15].

**Fig. 1:**
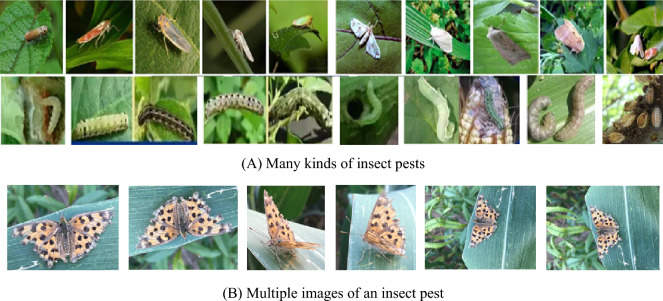
Insect pests in the field with various shapes, poses, sizes, colors, illumination and background

As shown in Fig. 1, the insect pest images taken in the field are diverse and irregular with different scales, shapes, poses, positions, illumination and complex backgrounds. Therefore, the existing crop insect pest detection methods are faced with some challenge, such as complex environment, detection of tiny size pest of multiple classes of pests, and the traditional ML algorithm is difficult to extract the invariant detection features, while the significant results of DL method rely on a large number of images and powerful computing power.

As for the various insect pests with deferent sizes, multi-branch, multi-channel and multi-scale DL models have been presented [16]. But these models have large trainable parameters, costing large training-time and computing ability. Dilated convolution can enlarge the receptive filed and improve the object detection ability of the network [17]. Several modified U-Nets did not consider the influence of U-Net combined with dilated convolution module on the result of feature extraction. Dilated ResNet can improve fine detection results [18]. Inception module can capture multi-scale context features by using multiple convolutional kernels of different sizes. It can not only capture long time–frequency context information of features, but also exploit information from multiple layers of CNN. Attention mechanism can help the network model locate the focus area, extract more useful features, and achieve high precision fusion. Attention based CNN has higher classification accuracy and significantly reduces the number of particles misclassified, which reflects the focusing effect of attention mechanism [19, 20].

As shown in Fig. 1, crop pest detection has some challenging issues that affect the accuracy of insect pest detection methods, such as multi-class, multi-scale, tiny size of pest objects, unbalanced data for multi-class, and sparse pest distribution. To improve the detection accuracy of field insect pests, an improved U-Net, namely the expanded multi-scale Attention U-Net (DMSAUN-Net), is constructed by using the advantages of ResNet, dilated convolution and Inception module. The main contributions of this paper are summarized as follows:

1. Dilated Inception module with various dilation ratios is introduced to extract the multi-scale contextual features.
2. Spatial attention mechanism is added to the skip connection layers of U-Net, which can focus the attention on the edge of the insect pest and reduce the noise and computational cost.
3. The computational cost is further reduced by introducing ResNet into the skip connection layer of U-Net.

The rest of this paper is organized as follows. The related works are summarized in "Related works". The proposed DMSAUN-Net based insect pest detection is illustrated in "Dilated Multi-scale attention U-Net (DMSAU-Net)". The detail experimental analysis and comparison is provided in "Experiments and analysis", and "Conclusions" summarizes this paper and points out the future work.

## Related works

In Section, U-Net, Inception module and Dilated convolution are briefly described. Inception module and Dilated convolution are widely introduced into DL model to improve its multi-scale objection detection performance.

### U-Net

**Fig. 2:**
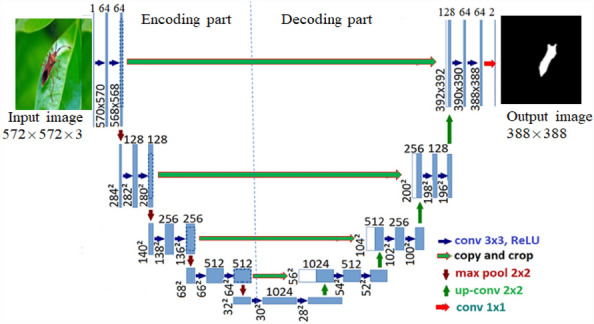
U-Net architecture

U-Net consists of encoding part, decoding part and skip connection without fully-connected layers. Its architecture is shown in Fig. 2. Encoding part is to extract high-resolution and contextual features with downsampling and one activation unit (ReLU) at each layer. Decoding part is to increase the resolution of the output through upsampling at each layer. Skip connection is used to fuse the features from the encoding part with the corresponding feature map of the encoding part, ensuring localization of the extracted contextual features. In the down-sampling process, the number of feature channels is doubled while is shrunk after deconvolution operation in up-sampling.

### Inception module

**Fig. 3:**
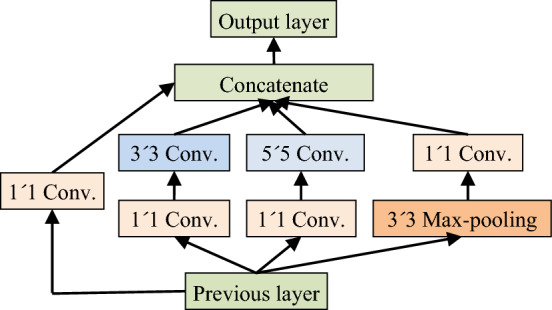
The structure of inception

Inception is a multi-branch multi-scale convolution module [21]. It can extract the multi-scale features from the input image by different-scale kernels. Its structure is shown in Fig. 3, including 1 × 1, 3 × 3 and 5 × 5 convolutional kernels, where 1 × 1 convolution operation is used to reduce the amount of calculation. To make the feature map have the same size, each branch adopts the same padding mode, and the stride is 1.

### Dilated convolution

**Fig. 4:**
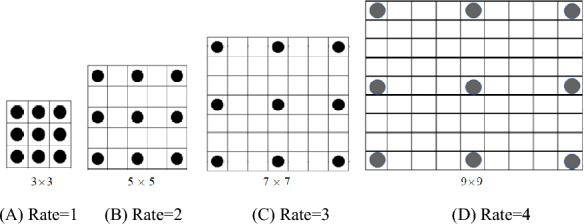
Dilated convolution with 4 dilation rates. **A** rate = 1. **B** Rate = 2. **C** Rate = 3. **D** Rate = 4

Dilated convolution can increase the receptive field without increasing the model parameters, which can reduce the computation amount and retain the nodal information [17, 18]. Its structure is shown in Fig. 4 with 4 dilated rates. It is seen from Fig. 4, the size of receptive field increases with the dilated rate, but the network parameters do not increase, that is 9 parameters.

Suppose an image *G* of size *m* × *n* and a convolutional kernel *W* of size *k* × *k*. The classical convolution between *G* and *W* is calculated by:

1 $$(G*W)(p) = \sum\limits_{i} {G[p + i] \cdot W[i]} .$$

Given a dilation rate* r*, dilated convolution (_∗*r*_) is defined as:

2 $$(G*_{r} W)(p) = \sum\limits_{i} {G[p + ri] \cdot W[i]} .$$

From Eqs. (1) and (2), it is evident that dilated convolution is simple convolution when *r* = 1. For *r* > 1, *r *− 1 zeroes are inserted between each kernel element, creating a $k_{s} \times k_{s}$ scaled and sparse filter, where $k_{s}$ is $k_{s} = k + (k - 1)(r - 1)$. The dilated rate *r* increases the receptive field of kernel by a factor $(k_{s} /k)^{2}$.

## Dilated multi-scale attention U-Net (DMSAU-Net)

**Fig. 5:**
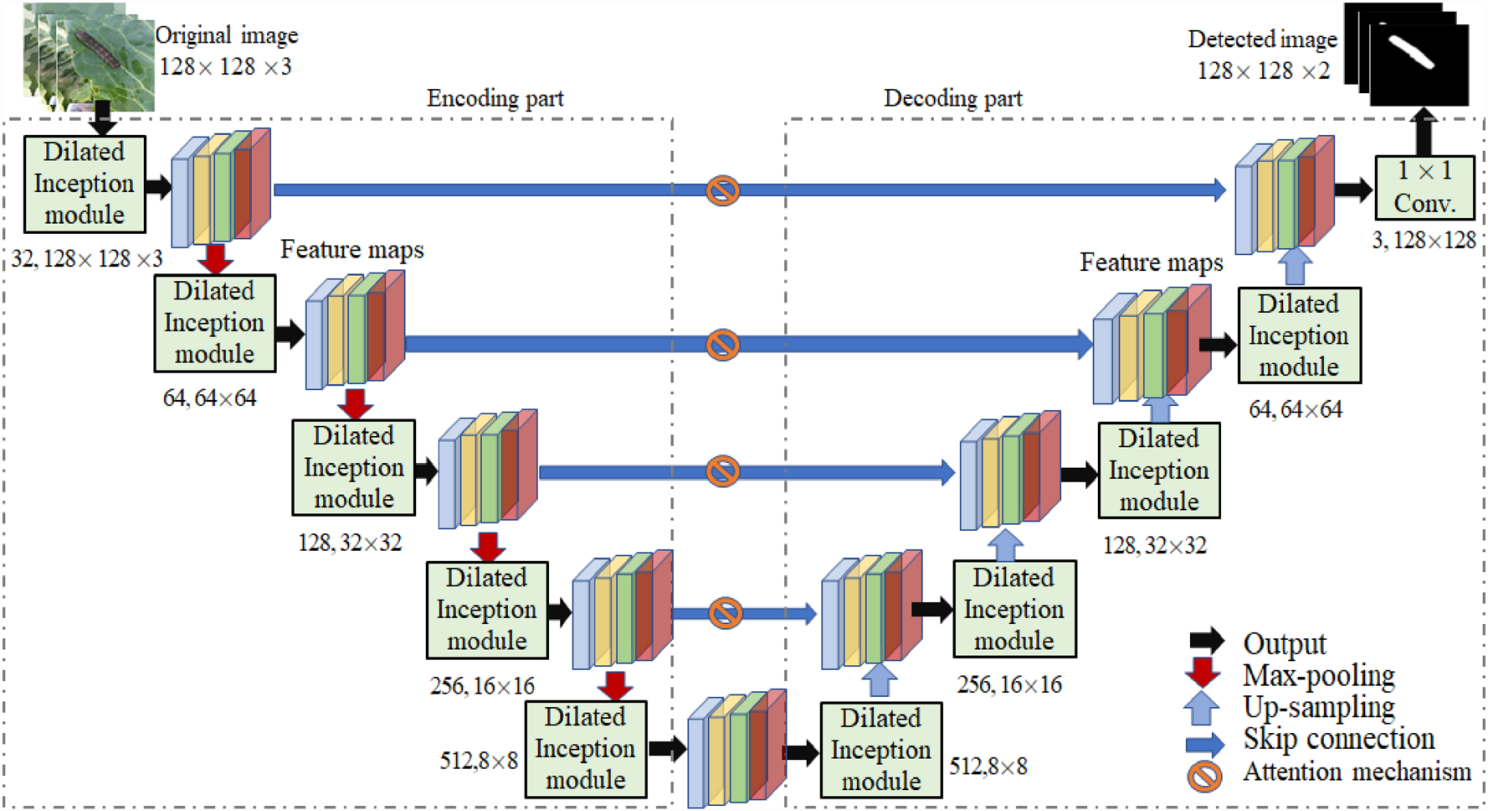
The structure of DMSAU-Net

Due to the small dataset and the easy influence of complex background such as illumination and clutter, as shown in Fig. 1, the detection accuracy of crop insect pest is low, which is over-detection or under- detection. In this Section, an improved U-Net model namely dilated multi-scale attention U-Net (DMSAU-Net) is constructed for insect pest image detection. Its overall structure is shown in Fig. 5. The numbers shown below each dilated Inception module indicate the total number of kernels used, height, width and depth of the output feature maps.

### Detail of DMSAU-Net

Similar to U-Net, DMSAU-Net mainly consists of the encoding part, decoding part and skip connection with attention mechanism. Encoding part is a multi-scale convolutional network, including dilated Inception module (convolution kernel of 3 × 3 kernel), the pooling layer (2 × 2 maximum pooling), and the activation function (ReLU). In encoding part, the multi-scale and multi-level features are extracted from images through dilated Inception module, and then the extracted features are downsampled to realize the correlation of multiple channels and the full decoupling of image feature space. In decoding part, the extracted features are restored by up-sampling (deconvolution of 2 × 2), skip connection with attention, dilated Inception module (convolution kernel of 3 × 3) and activation function (ReLU) classification. Finally, the binary insect pest image of the insect pest and the background is obtained by 1 × 1 convolution layer and Sigmoid activation function. Considering the possible mesh effect caused by deconvolution, the bilinear interpolation method in upsampling is used to restore the image, during which 1 × 1 convolution is used to restore the number of channels. During the up-sampling process, the feature maps corresponding to the same resolution of down-sampling are concatenated. After each concatenation, the feature maps are further refined through dilated multi-scale module, and the upsampling is performed successively until the features extracted from the encoder are restored to the size of the input maps. Skip connection with attention is to concatenate the convolutional features and the deconvolution features.

### Dilated inception module

**Fig. 6:**
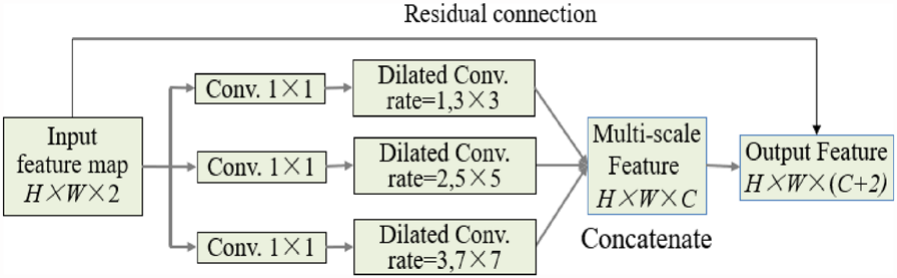
The structure of dilated inception module

Inspired by Inception module, multi-scale concatenation module and dilated concatenation module, a dilated Inception module is constructed as shown in Fig. 6, consisting of 3 1 × 1 convolution kernels, 3 dilated convolutional kernels, a concatenation, and residual connection. It is a modified Inception module, which aims to extract multi-scale features, from low-level structural features to high-level semantic features, by increasing receptive field without increasing the training parameters.

Given an input *G*_*or*_, from Fig. 6, the dilated Inception module can generate multi-scale features denoted as *G*_*i*_ (1 ≤ *i* ≤ 3) by employing three convolutions to collect contextual data at various scales. The convolution layer with kernel size of 1 × 1 is utilized to reduce the calculation cost. Since the multi-scale features of the 3 channels *G*_*i*_ are independent of each other, the transmission of global contextual features is limited. To address this problem, the global average pooling layer is used to provide richer contextual features *G*_gap_:

3 $$G_{{{\text{gap}}}} = Up({\text{conv }}(gap(F))),$$

where *Up* is a bilinear interpolation operation to up-sample contextual features to the same size as *G*_*i*_, *gap* is the global average pooling, and *conv* is a 1 × 1 convolutional operation.

The multi-scale features *G*_*i*_,* G*_gap_ and *G*_*or*_ are combined to obtain the output feature *G*:

4 $$G = {\text{ conv }}\left( {G_{{1}} + G_{{2}} + G_{{3}} + G_{{{\text{gap}}}} + G_{or} } \right) ,$$

where the convolution process 1 × 1 is calculated by *conv* and the concatenation by ‘+’.

### Attention module

**Fig. 7:**
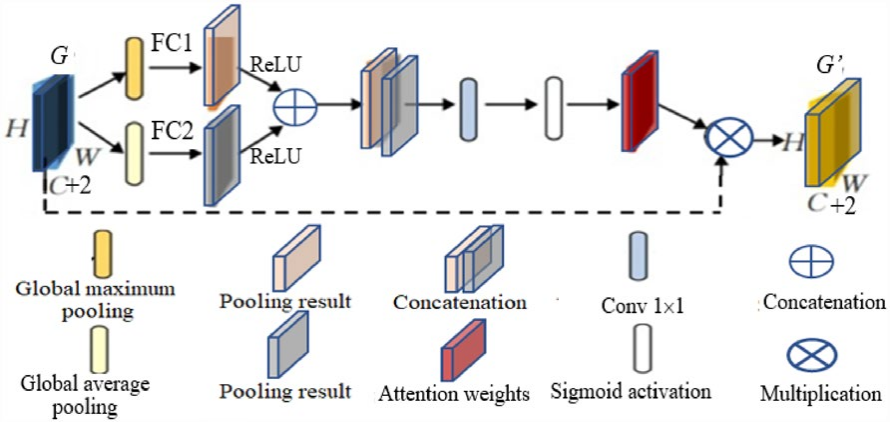
The structure of attention module

Dilated Inception module obtains multi-scale features by encoding part and decoding part, and the extracted multi-scale features are concatenated by skip connection to achieve more accurate details and location information of insect pest. However, the max-pooling and upsampling operation in the encoding and decoding parts will lose part of the location space and other information, resulting in inaccurate segmentation of insect pest in the field. To overcome this problem, the spatial attention mechanism module is added to Skip connection. By directly cascading the features of the encoding layer and the corresponding deconvolution layer, the attention module fuses their complementary features, suppressed the noise generated by upsampling, and enhanced the robustness of the model [17]. The structure of attention module is shown in Fig. 7.

In attention module in Fig. 7, the input feature map* G* ∈ *R*^*H*×*W*×(*C*+2)^, is the output of dilated Inception module in Fig. 6, is fed into the global maximum pooling (GMP) layer and global average pooling (GAP) layer, respectively, obtain *G*_gmp_ ∈ *R*^1×1×(*C*+2)^ and *G*_gap_ ∈ *R*^1×1×(*C*+2)^, are fed into the two fully connection layers *Fc*_1_ and *Fc*_2_ to get the output *G*_*max*_ and *G*_*avg*_ in each branch. The number of parameters is decreased since the parameters of *Fc*_1_ and *Fc*_2_ are shared by two channels. The process is a detailed calculation:

5 $$G_{\max } = {\text{ReLU}}\left( {Fc_{{1}} \left( {G_{{{\text{gmp}}}} } \right)} \right),\; G_{avg} = {\text{ReLU}}\left( {Fc_{{1}} \left( {G_{{{\text{gap}}}} } \right)} \right).$$

Then the attention weight *S*_*a*_ by sigmoid activation function is generated as follows:

6 $$S_{a} = {\text{ Sigmoid}}(G_{\max } + G_{avg} ).$$

To obtain the attention map *G*_*a*tt_, the multiplication operation of the features *G* and the attention weight *S*_*a*_ is required. The attention map *G*_*a*tt_ is calculated as follow:

7 $$G_{{a{\text{tt}}}} = G \times S_{a} .$$

### Loss function

The weighted IoU loss function *L*_*IoU*_ and the weighted binary cross entropy loss function *L*_*bce*_ are used to construct the loss function *Loss* in DMSAU-Net, calculated as follows:

8 $$Loss = L_{IoU} + L_{bce} .$$

To achieve effectively crop insect pest detection, the two loss functions are used to represent the global and local supervision losses, respectively.

### Model training and evaluation

The input insect pest images and their corresponding labeled images are used to train DMSAU-Net. Five*-*fold cross-validation (fivefold CV) scheme and stochastic gradient descent (SGD) with an adaptive moment estimator (Adam) are often used to train all models [22].

The purpose of crop insect pest detection is to determine the category of each pixel in the image, so as to clarify the scope of insect pests. Accuracy and Intersection over Union (IoU) are selected as indexes to evaluate the segmentation performance of the proposed algorithm, calculated as follows:

9 $$ACC = \frac{TP}{{TP + FN}},\;\;\;IoU = \frac{TP}{{TP + FP + FN}},$$

where *TP*, *FP* and *FN* are the numbers of true positives, false positives, and false negatives of the class, respectively.

## Experiments and analysis

To validate the proposed DMSAU-Net based insect pest detection method, the insect pest detection experiments are conducted on the crop common insect pest image dataset, compared with two insect detection approaches: multi-scale super-resolution feature enhancement module (MSR-RCNN) [6], multi-projection pest detection model (MDM) [7], and compared with U-Net [23] and its two improved models: U-Net with dilated convolution (DCU-Net) [24], ResNet with U-Net (ResU-Net) [25]. Batch size = 32 for rice data subset of IP102 to reduce computation time. Number of iterations = 3000, global learning rate = 0.001, gradient decay factor = 0.9, squared gradient decay factor = 0.999, loss function = cross entropy. All models are tested on Keras and trained on an Intel Xeon E5-2643v3 @3.40 GHz CPU, GTX2080Ti 11 GB GPU, 64 GB RAM, Windows 7 64bit, CUDA Toolkit10.0, CUDNN V7.6.5, Python 3.7 and Tensorflow-GPU 1.8.0.

### insect pest image dataset

**Fig. 8:**
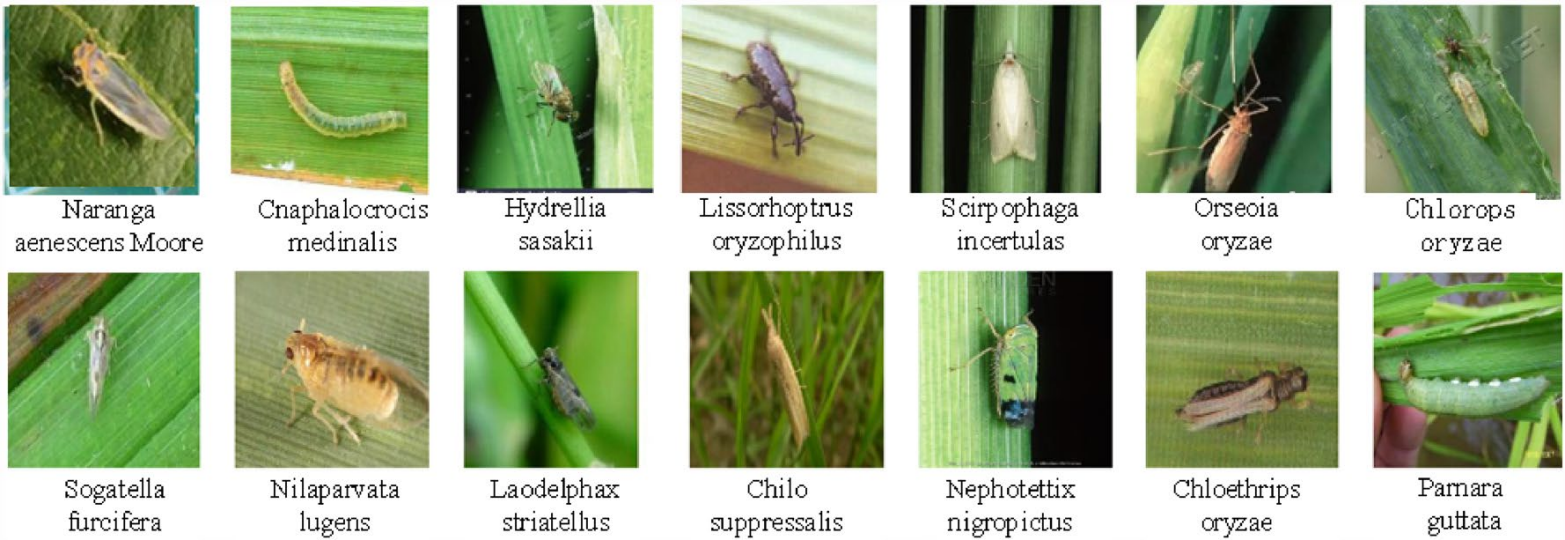
Fourteen original insect pest images, one per species

IP102 (https://github.com/xpwu95/IP102) is a public insect pest image dataset, containing more than 75,000 images belonging to 102 insect pest categories that exhibit a natural long-tailed distribution [26]. 19,000 of these images have be professionally annotated. There are 8415 insect pest images in the dataset belonging to 14 rice insect pest categories, as shown in Fig. 8 and Table 1. From Table 1, it is seen that the classes of rice insect pests are highly unbalanced, ranging in sample size between 173 and 1115. In the experiments on this data subset, five-fold-cross validation scheme is adopted to perform experiments. That is, the dataset is randomly split into *5* mutually exclusive subsets of equal or near equal size. The model is performed 5 times subsequently, where each time using 4 of the 5 splits as the training set to train the model, and the 1 of the 5 splits as the test set to evaluate the performance of the model. To verify the robustness of the proposed method, images under different conditions such as strong illumination and complex background are selected, and the same insect pest in the dataset contained different insect states. To improve the recognition accuracy, Photoshop is used to uniformly adjust the image to 128 × 128 pixels.

**Table 1:** Rice insect pest image dataset

No.	Insect pest name	Amount	Number
1	Rice leaf roller	1115	0–1115
2	Rice leaf caterpillar	485	1116–1601
3	Paddy stem maggot	261	1602–1862
4	Asiatic rice borer	1053	1863–2915
5	yellow rice borer	504	2916–3419
6	Rice gall midge	506	3420–3925
7	RICE Stemfly	369	3926–4294
8	Brown plant hopper	838	4293–5132
9	White backed plant hopper	889	5133–6021
10	Small brown plant hopper	553	6022–6574
11	Rice water weevil	856	6575–7430
12	Rice leafhopper	404	7431–7834
13	Grain spreader thrips	173	7835–8007
14	Rice shell insect pest	409	8008–8416

### Experimental results

**Fig. 9:**
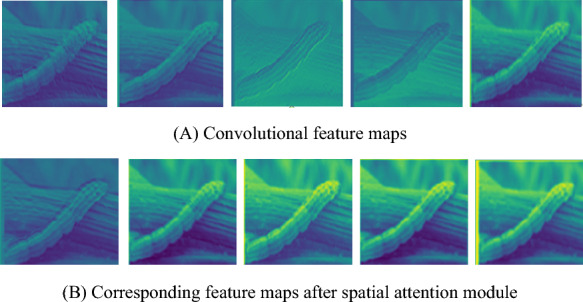
Convolutional feature maps

To test the effectiveness of spatial attention module, Fig. 9 show the convolutional feature maps of the first dilated multi-scale module and the corresponding feature maps after spatial attention module. From Fig. 9, it is obvious that the convolutional feature maps after spatial attention module are more significant than that of the first dilated multi-scale module.

**Fig. 10:**
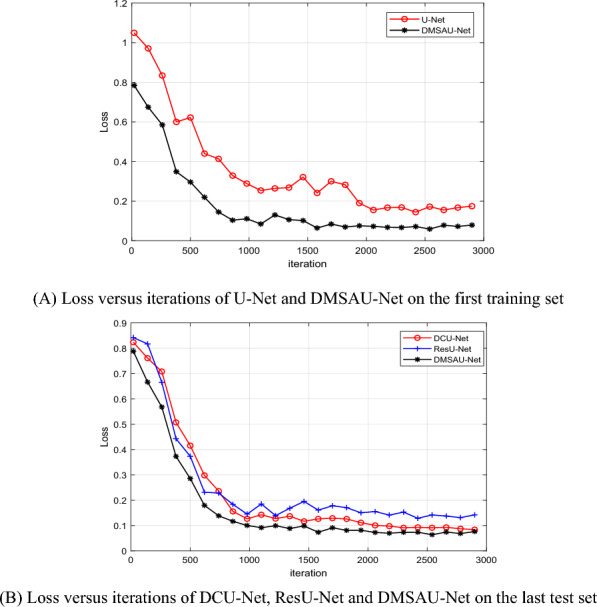
Loss versus iterations

DMSAU-Net is a modified U-Net, is similar to DCU-Net and ResU-Net in structure. So, we compare the performances of U-Net, DMSAU-Net, DCU-Net and ResU-Net. Figure 10A shows the loss values versus the number of iterations of U-Net and DMSAU-Net on the training set. From Fig. 10A, it is found that DMSAU-Net converges much better than U-Net.

To further analyze the training performance, the same experimental parameters are used to train the different networks to ensure the reliability of the comparison results. The detection performances of DMSAU-Net, DCU-Net and ResU-Net are shown in Fig. 10B. From Fig. 10B, it can be seen that as the number of iterations increases, the detection loss of the three models decreases, and the detection performance of DMSAU-Net outperformances other models. Before 1000 iterations, their losses decrease quickly, go down very slowly and then level off. From Fig. 10, it can be seen that their training processes are relatively stable after 2000 iterations, DCU-Net and DMSAU-Net are better than ResU-Net, and DMSAU-Net is better than DCU-Net. This owes to the dilated multi-scale convolution and spatial attention mechanism.

**Fig. 11:**
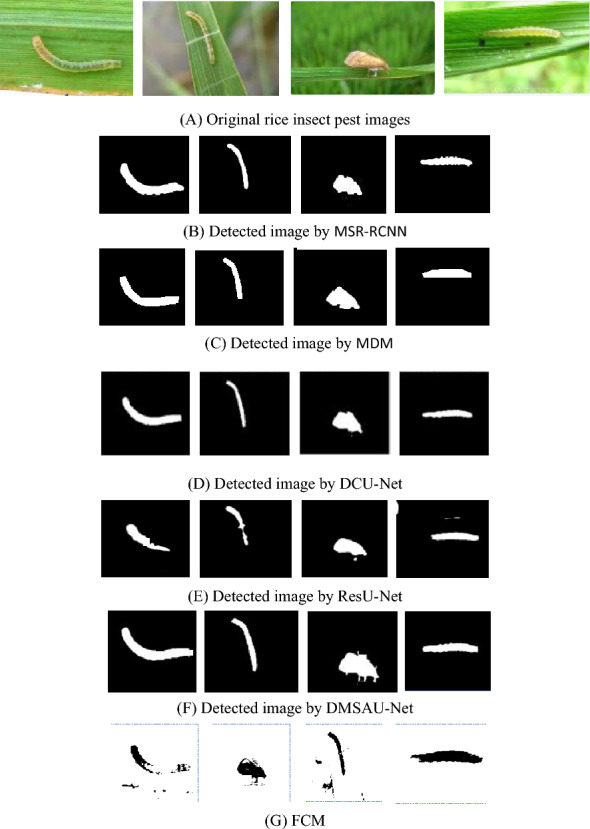
Rice insect pest detection of IP102

To test the detection performance of DMSAU-Net, we randomly select 4 rice insect pest images from the IP102 dataset and visualize the detection results of DMSAU-Net and 4 comparative approaches, as shown in Fig. 11. To reflect the advantages of deep learning methods, we compare DMSAU-Net with a traditional insect pest detection algorithm Fuzzy C-means (FCM). It is one of the most widely used methods of insect pest detection [27]. The detection results are also in Fig. 11. As can be seen from Fig. 11, 5 improved U-Net models are much better than FCM. They can effectively detect rice insect pests under complex background, and the position and shape of insect pests are good, among which, MSR-RCNN is better than the other comparative models, DCU-Net is better than MDM and ResU-Net. Overall, DMSAUN-Net has the best detection effect with more accurate insect pest shape and edge.

To quantitatively estimate the detection performance of DMSAU-Net, fivefold cross validation experiments are conducted on the rice insect pest image subset of IP102. As can be seen from Fig. 10, all models basically converge at the 3000th iteration. For fair, the trained models are selected at the 3000th iteration. The detection results of five comparative methods and DMSAU-Net are listed in Table 2.

**Table 2:** The detection results of rice insect pests by 4 insect pest detection algorithms

Results	Method					
	U-Net	MSR-RCNN	MDM	DCU-Net	ResU-Net	DMSAU-Net
Accuracy (%)	84.2	90.8	87.9	90.7	88.5	94.1
IoU (%)	81.4	89.7	84.6	89.2	86.3	91.2
Training time (h)	15.6	10.5	11.4	9.5	8.3	7.7
Test time (ms)	51	47	37	45	38	31

DMSAU-Net is constructed by making use of the advantage of U-Net, dilated convolution, multi-scale convolution and spatial attention mechanism. To further verify the superiority of DMSAU-Net, some ablation experiments are carried out under similar conditions. The different experimental set and rice insect pest results are shown in Table 3, where attention is added to skip connection of U-Net.

**Table 3:** The different experimental set and results

Experimental set	Results		
	Accuracy	IoU	Training time (h)
U-Net	84.2	81.4	15.6
U-Net + spatial attention	85.6	84.3	13.2
U-Net + Inception	87.2	86.4	13.7
U-Net + dilated multi-scale	88.1	87.5	11.0
U-Net + Inception + spatial attention	89.0	87.9	10.7
U-Net + dilated multi-scale + channel-spatial attention	94.16	91.24	8.6

Table 3 indicates that spatial attention, channel-spatial attention, Inception, dilated multi-scale can contribute to the results to some extent.

### Analysis

From Figs.10 and 11 and Tables 2 and 3, it can be seen that DMSAU-Net has best detection performance, the highest detection rate and the least training and detecting time, due to dilated convolution, multi-scale convolution and spatial attention mechanism. With the aid of dilated multi-scale, DMSAU-Net can extract the multi-scale classification features. With the aid of skip connection combined with spatial attention mechanism, DMSAU-Net can enhance the constraint on the feature maps, focus more attention to the insect pest image region, and speed up the training. That is to reduce the learning of non-important areas and enhance the learning of insect pest areas, so as to improve the detection ability of insect pest characteristics and improve the detection accuracy rate. The detection rate of U-Net is the poorest, because it is difficult to extract the robust classification features from the various insect pest images with very complex background.

## Conclusions

In modern agricultural field, insect pest detection plays an important role in timely and accurate diagnosis of crop insect pest. But it is difficult to detect crop insect pest in the field due to the various-shape-size insect pests with complex background. To solve such problem, a dilated multi-scale attention U-Net (DMSAU-Net) model is constructed for crop insect pest detection by making use of the advantage of multi-scale convolution and attention mechanism. In dilated inception module, multi-scale convolution kernels without increasing training parameters are used to extract the distributed characteristics of insect pests at different scales and to perform cascade fusion. The experiments are carried out on the rice insect pest image subset of IP102 dataset. The detection results show that DMSAU-Net is effective and feasible for crop insect pest detection in the field. This research can be used to realize the automation degree of insect pest management in agricultural field. Future work is to optimize the model to organically integrate it into an effective insect pest detection system.

DMSAU-Net is complex in structure and has many data processing processes, and is difficult to detect the occlusion insect pest in field, which is a common phenomenon. In the future, we will prune and optimize the model, continue to study how to improve the accuracy of insect pest detection and the generalization ability of the model, and construct a multi-scale feature fusion DMSAU-Net to deal with the occlusion problem.

## Data Availability

The data that support the findings of this study are openly available in https://github.com/xpwu95/IP102.
